# Hybrid Inspired Research on the Flying-Jumping Locomotion of Locusts Using Robot Counterpart

**DOI:** 10.3389/fnbot.2019.00087

**Published:** 2019-10-23

**Authors:** Dunwen Wei, Tao Gao, Zhaoxin Li, Xiaojuan Mo, Shuqin Zheng, Cong Zhou

**Affiliations:** ^1^School of Mechanical and Electrical Engineering, University of Electronic Science and Technology of China, Chengdu, China; ^2^Faculty of Engineering, The China University of Petroleum-Beijing at Karamay, Karamay, China; ^3^School of Mechanical Engineering, Northwestern Polytechnical University, Xi'an, China

**Keywords:** hybrid inspiration, bioinspired robots, robotics-inspired biology, multi-modal locomotion, flying-jumping locomotion, locust

## Abstract

Locusts are a kind of agile insects that can move and maneuver so efficiently in the unstructured terrain and complex environment. This marvel survivability of locusts benefits from their flying-jumping multi-modal locomotion. But until recently, the main influences of the locomotion performance are still a controversial and unknown issue. In this paper, the idea of hybrid inspired method that combines biologically inspired robot with robot inspired biology was proposed to explore the principle of flying-jumping locomotion of locusts. Firstly, we analyzed the influence of leg burrs and flapping wings on the jumping performance by the biological experiments. Nevertheless, individual heterogeneity and uncontrollability of locusts result in the unconvincing results of biological experiments. Therefore, according to the thought of robotics-inspired biology, we proposed and built a locust-inspired robot with flying-jumping locomotion via the principle of metamorphic mechanism based on the biological-inspired robot. Lastly, the preliminary robotic experiments were carried out to validate our thought that the flapping wings and leg burrs of locust have a great influence on the jumping performance. This robotics-inspired biology method remedied the shortcomings of biological experiments through the consistency and controllability of the robot experiments. Meanwhile, through the hybrid inspired research, the results show both the leg burrs and flapping wings can help the locust jump longer and improve the stability by adjusting the landing attitude to some extent, while the biological experiments dedicate that the locust with leg burrs and wings have the self-stability ability.

## Introduction

The inspiration of biology as a well-known method has led to the design of biologically inspired robots (Meyer and Guillot, 2008). This category robots are also commonly called bioinspired robots (Iida and Ijspeert, 2016). Many bioinspired robots are inspired by the locomotion of animals to move and manipulate so effectively in their environment (Lu et al., 2018). But until recently, the main influences of determining the locomotion performance of different animals in different complex environments are still a controversial and unknown issue (Peyré-Tartaruga and Coertjens, 2018). The conventional method by biological experiments is the mainstream of exploring the locomotion principle of animals. The challenges of biological experiments are to snapshot the focused locomotion state and keep the repeatability of experiments. However, because of the individual heterogeneity and uncontrollability of animals, the biological experiments result in the irregular, or unconvincing results. Therefore, the robot experiments are serving as an advanced replacement for the biological system to explore and discover the principles or mechanisms behind the biology (Karásek et al., 2018). This method of providing new scientific knowledge for biology is named as robot inspired biology (Gravish and Lauder, 2018). Biologically inspired robot and robot inspired biology are the cross-disciplinary research frontiers of engineers and scientists in the future of robotic and biological locomotion research (Romano et al., 2019).

In this paper, the idea of hybrid inspired research that combines biologically inspired robot with robot inspired biology was proposed and adopted to explore the principle of locust flying-jumping locomotion. According to the idea of the biologically inspired robot, we conducted the biological experiments for initially exploring the flying-jumping locomotion of locusts. Such biological experiment provides the idea for developing one locust-inspired flying-jumping robot. According to the idea of the robot inspired biology, we explored the influence of leg burrs, and flapping wings on the jumping performance by robot experiments. In the following parts, we first reviewed the state of arts of locust flying-jumping locomotion research from both locust biology aspect and locust-inspired robot aspect before we continue our hybrid inspired research.

From the biological perspective of locusts, it is well-known that the locusts are a kind of agile insects capable of performing multi-modal locomotion with both flying and jumping to efficiently navigate in the unstructured terrain and complex environment (Zaitsev et al., 2015). On the one hand, locusts take the typical jumping locomotion as the main physical strategy to travel rapidly on the ground and manipulate different obstacles. Benefiting from the long hind legs with enlarged femora, locusts have a greater mass of extensor tibiae muscles and have a long stretching distance for acceleration. On the other hand, because of the long period of biological evolution, locusts have evolved the jump to launch into flight, and the flying locomotion with wings is a means of rapid escape. The research about the jumping behavior and adopt a catapult mechanism (Burrows, 2016), in which of the hind legs contract to store energy in distortions of the exoskeleton, and then suddenly release the energy to power the hind legs' rapid movement. This flying-jumping locomotion has the advantage of overcoming large obstacles in unstructured terrains comparing with the other wheeled and crawling locomotion. And because of that, the jumping locomotion via the locust biological experiment has been studied for several decades, starting from the 1970s with some distinguished work (Camhi, 1970; Bennet-Clark, 1975; Heitler and Burrows, 1977). In recent years, Chen et al. (2015) examined the mechanism of air posture adjustment of locusts and declared that the wings and abdomen are mainly utilized for air posture adjustment. Mo et al. (2019) studied the influence of substrate roughness on the jumping performance and claimed that there was no significant relation between ground roughness and jumping success. In order to improve the accuracy and efficiency of biological experiments, Zhou et al. (2019) developed a jumping locomotion detection system named JumpDetector with automatic trajectory tracking and behavior analysis to evaluate the endurance jumping of locusts.

Despite decades of study, little is known about how leg burrs and flapping wings influent the jumping performance. However, in nature, the complexity of leg burrs and flapping wings interaction with the ground and air often rivals or even exceeds that of theoretical analysis. But an advanced understanding of leg-wing interaction and flying-jumping locomotion in complex terrain is relatively lacking. To overcome this challenge, we firstly performed the flying-jumping locomotion studies of how the locomotion performance depends on leg burrs, flying wing, and leg morphology.

In the aspect of the research of locust-inspired robot, multi-modal locomotion robotic systems are new concepts of bio-inspired robotics with two or more of locomotion (Low et al., 2015; Mintchev and Floreano, 2016). Such bio-inspired robotic systems may lack direct connectivity to their biological counterparts, but are useful for understanding the principles of mechanical design and control as well as for understanding the principles behind the biology. In the recent years, there are some proposed robots with the flying-jumping multi-modal locomotion (Fukuda et al., 2012). Inspired by the flying and jumping behaviors of the desert locust, Beck et al. (2017) recently developed a miniature jumping robot with spreading wings and a tail to assistant jump over 1.7 m. Woodward and Metin (2014) and Woodward (2017) built a biologically inspired jumping-gliding robot, named as Multi-bat, whose mass is only 100 g. This robot has two four-bar mechanisms flapping wings which can make the robot jump over meters. In order to increase the jump gliding distance and reduce the landing impact energy, Kovac et al. (2008); Kovač et al. (2011) designed a jump gliding mechanism and installed it on the small locust jumping robot. Li et al. (2012) improved the previously developed jumping robot Grillo (Scarfogliero and Stefanini, 2006), added the wings to increase the jumping distance and hovering time, and the tail swinging from left to right can adjust the gliding direction. Chen et al. (2015) designed an air attitude adjustment system for a locust-inspired robot through the asymmetric movement of wings to verify the mechanism of attitude adjustment. It proved that locusts adjust their attitude in the air through the asymmetric movement of their wings.

The current research mainly designed the static wings on the basis of jumping robot to enhance the stability during the takeoff, flight, and landing. However, the static wing can only increase the gliding distance but not provide enough lift force. Zhang et al. (2017) proposed a multi-modal locomotion robot with jumping-flapping locomotion to study the feasibility of jumping aided takeoff, but it is only a conceptual design. Therefore, it is necessary to add flapping-wing on the basis of jumping locomotion, and to develop a locust-inspired robot with flying-jumping multi-modal locomotion, therefore the robot can use the jumping motion to take off the ground, and use the flapping wings to fly after taking off, then it can flap wings slowly to buffer the impact when landing. In nature, there are many insects that have similar locomotion modes like this. The locust is one of the insects that have both the flying and jumping locomotion. However, the influence of locust's leg burrs and flapping wings on jumping performance, the factors affecting the jumping performance and how the factors affect the jumping performance of locusts are still unknown. So, inspired by the locomotion of locusts, we built a flying-jumping bioinspired robot. We validated the deduction drawn from the biological experiment upon locusts by the robot prototype based on the idea of robotics-inspired biology.

Our contribution in this paper is to bridge this gap by the method of hybrid inspired research to discover the influence factors of locomotion performance, and use biological experiments and robot experiments to better understand biological movement and advance robotic mobility in the real world. On the aspect of biological experiments, we studied the animals' biology and locomotion behavior that influences the flying-jumping performance through some unique leg and/or wing surgeries for living locusts. On the aspect of robot physical experiment, we inspired from the locomotion of locusts to design and build new locust-inspired robots with the improved locomotor capabilities. Based on the locomotion mechanism of the locust legs coupled with its wings, the jumping mechanism and the flying mechanism were, respectively, designed and analyzed using the abundant force and trajectory characteristics of the gear-bar mechanism. Combined with the principles of metamorphic mechanism, the flying-jumping mechanism was designed by coupling the flapping-wing mechanism with the jump mechanism. We used robots as physical models to test hypotheses of the flying-jumping locomotion. These robots enable control and variation of influent parameters and allow precise, repeatable, and systematic experiments. The use of bio-inspired robots as physical models, rather than locust animals, provides a few advantages in studying the complex locomotion: (a) Biological experiments on locust animals can disrupt their lives and raise ethical questions, involving caging them and, if necessary, manipulating, or operating on them. (b) Biological experiments with animals vary from one individual to another and from one trial to another, and thus generate data with large variability, while robots are simpler than animals, with fewer degrees of freedom and more well-defined morphology and kinematics. Robots are much more repeatable. (c) Robots can often be modified more easily and allow easier parameter variation, whereas animals can be hard to modify without affecting their behavior.

This paper is arranged as follows. In section Locomotion Principle From Biological Experiments, we firstly investigated the influences of both the leg burrs and flapping wings on jumping performance by biological experiments, and then we made some biological deductions based on the biological experiment results. In section Design of Locust-Inspired Robot, we proposed one locust-inspired robot, which has flapping and jumping multi-modal locomotion based on the principles of the metamorphic mechanism. By the thought of robot inspired biology, we carried out the preliminary experiments to validate our thought that the flapping wings and leg burrs of locust have a great influence on the jumping performance.

## Locomotion Principle From Biological Experiments

Biological experiments on the characteristic and locomotion of locusts can accurately grasp the structural parameters and flying-jumping locomotion. The coupling principle of leg burrs, leg bounce, and flapping wings is the main factor that affects jumping performance. Therefore, exploring the mechanism between the leg and the flapping-wing are of great significance for the study of locomotion performance. In this section, the possible influencing factors, such as the number of leg burrs, the bounce legs, and the flapping-wing on the jumping performance are observed experimentally, and the mathematical model is used to calculate the influence on the jumping performance, to find out the factors affecting the jumping performance of the locusts, and to reveal the locust legs. The mechanism of motion coupled with the wing provides a theoretical basis for the study of robots with flying-jumping locomotion.

The mechanism of bionic locomotion is the basis and key point of research on the flying-jumping locomotion of locusts. From the perspective of biophysical structure, it is the inevitable trend of structural bionics. Based on the enlightenment from biological take-off, flight, and landing behavior, we explore the factors affecting the jumping performance of locusts and reveal the mechanism of stability affecting the takeoff, flight, and landing of locusts.

In order to study the influence of leg burrs and flapping wings of locusts on jumping performance, we conducted some biological experiments using one kind of wild east Asian migratory locusts living in China and analyzed their jumping and flying behavior. By observing the influence of the number of burrs in the legs, the flapping wings and the coupling of leg bouncing and flapping motion on the jumping performance, we try to find out the main factors that affect the jumping performance and reveal the mechanism of the coupling movement between the legs and wings.

### Experimental Procedures of Locomotion

**Figure 1:**
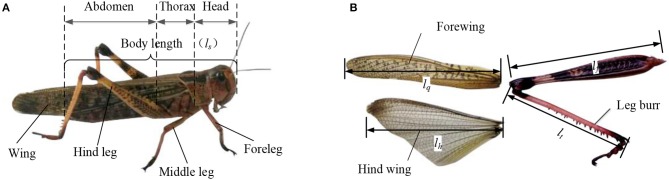
The morphological characteristics of locusts **(A)** the body structure of locust consisting the head, chest, abdomen, wings, and legs; **(B)** flapping-wing and leg structure of locust.

As shown in Figure 1A, the body structure diagram of locust consists of five parts, which are the head, the thorax, the abdomen, the wings, and the legs. Six legs are symmetrically distributed on both sides of the central axis of the body, which are two forelegs, two middle legs, and two hind legs. As shown in Figure 1B, the hind legs with many leg burrs are thicker and stronger than the forelegs and middle legs, which is good for the jump. Compared with the strong hind leg to promote the force, the forelegs and the middle legs are mainly used for crawling to provide the support force and the ground cushioning. The structure of flapping wings includes the forewings and hind wings, in which the leather of forewings is covered on the hind wings when not flying. The forewings, also known as the tegmina, have the tough wing veins and cover on the back of the body during crawling and protects the hind wing. When flying, the forewings can increase the contact area with air and increase the lift. The wing chord of hind wings is longer than the forewings. The texture of hind wings is soft and easy to deform. The hind wing, with film-like leather driven by the chest muscles, can be folded and shrunk.

The possible influencing factors, such as the number of leg burrs, the bounce legs, and the flapping-wing on the jumping performance are observed by experiments. The whole jumping performance of locusts is captured by cameras. The influences of different wing lengths and the number of leg burrs on the jumping performance are observed and analyzed. The biological experiment consists of the following five steps, namely, preparation, treatment, recovery, testing, and post-processing.

#### Preparation

**Figure 2:**
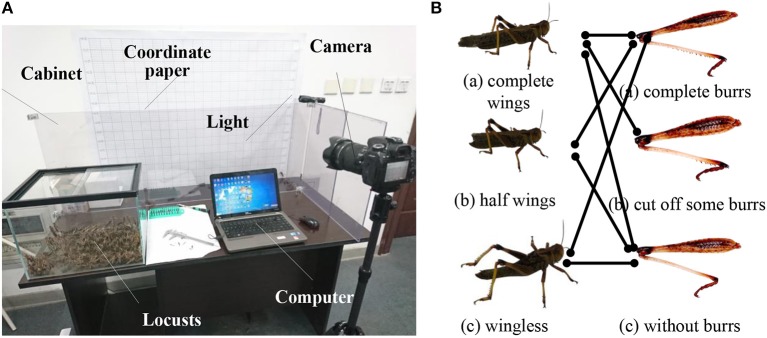
Biological experiments **(A)** experimental setup; **(B)** group of different treatments for flapping wings and leg burrs.

In the preparation step, as shown in Figure 2A, in order to eliminate the influence of individual differences in biological experiments, we chose east Asian migratory locusts with similar body parameters (4~5 cm body length), strong physique and complete limbs. The candidate locusts were feed with enough food and reared at a cuboid glass container with about 22 W light and 30 ± 2°C temperature.

#### Treatment

In order to study the effect of leg burrs and flapping wings on jumping performance, we conducted some treatments for the candidate locusts. This is probably the most delicate experimental process step by manual. We must manually operate the candidate locusts to the leg burrs and/or flapping wings. As shown in Figure 2B, we carried out the different operations for both flapping wings and leg burrs, respectively. For the treatment of the wings, we divided them into three groups; (a) normal locusts without any treatment for flapping wings; (b) the locusts with half of the wings being cut off; and (c) the locusts without wings. Similarly, we conducted some treatments for leg burrs under the same external conditions. We obtained three candidate groups; (a) locusts with the complete leg burrs and without any treatment; (b) locusts with some leg burrs being cut off; and (c) locusts with all burrs being cut off. The experiment conditions and experiment environment are the same as those in the wings experiment. After the treatment, seven groups of candidates were obtained and denoted by the connection lines shown as Figure 2B.

#### Recovery

Because the treatments for candidate locusts will more or less affect their behavior, it is hard to make the locusts we chose to perform equally without variability. As a result, we made the following efforts to make the experiment data more authentic. In order to make the locust in each group have the unique variable, we let the locusts have enough rest and enough time to recover after operation treatment such as cutting off the leg burrs and/or wings. During the period of resting and recovering, we fed them with the equal amount of food, and make them live in the appropriate environment to keep them in a well-health condition and reduce the influences of irrelevant variables and inconsistent condition.

#### Testing

In the experiment, we use the high-speed camera to record the motion trajectory. Meanwhile, we use the scale as a background to calculate jumping height and distance directly. In order to obtain more accurate experiment data, we tried our best to shorten the duration of the experiment. We also add a group of locusts that jump on the oily ground as a contrast, in which the locusts only slipped on the ground and couldn't perform any jumping. In order to reduce accidental error, we let the locusts in each group perform 10 times jumps and recorded the average value of the jumping height and the jumping length in each group.

#### Post-processing

The video files were input into MATLAB software, and the jumping performance of different groups was recorded. The locomotion parameters of the jumping behavior were analyzed on the basis of the experiment data.

### Result Analysis of Biological Experiments

From the experiments, we found that the locust stores energy slowly and releases energy quickly through the structure of hind legs to jump and then fly by the flapping wings. There are four phases in its jumping process: leg contraction phase, take-off phase, flight phase, and landing phase. The first phase is to adjust the body posture by forelegs and middle legs, meanwhile, the hind legs constrict to store energy. Then the energy is released quickly in the take-off phase, phase to make the body take off the ground. In the third phase, the body moves forward through wing flapping motion. Then in the last phase locusts use slowly flapping motion to provide some lift and jump buffer of leg joints to reduce the contact impact force between leg and ground. Meanwhile, locusts use the leg burrs on tibia legs and appendages to increase the friction force between legs and ground, to prevent the tipping, slipping, or the rebounding that causes them bouncing off the ground when they are landing. The jumping and flying locomotion is mainly related to hind legs and wings, so we analyzed the leg burrs and wing integrity. By observing the effect of the number of leg burrs, the wing integrity, and the coupling of leg bouncing and wing-flapping motion on the jumping performance, we try to find out the factors that affect the jumping performance and reveal the mechanism of the coupling movement between the legs and wings.

**Figure 3:**
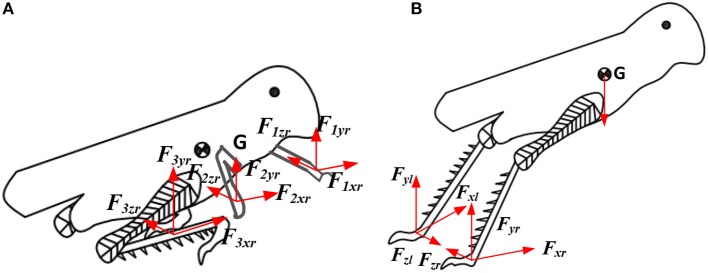
Schematic diagram of locust jumping model. **(A)** Leg contraction phase; **(B)** the take-off phase.

In the leg contraction phase, as shown in Figure 3 the force expression of the locust model is shown in the following equation:

(1) $$\left\{ \begin{array}{l} \sum_{i=1}^{3}F_{ixr}+\sum_{j=1}^{3}F_{jxl}=m\overset{▪▪}{x}\\ \sum_{i=1}^{3}F_{iyr}+\sum_{j=1}^{3}F_{jyl}-mg=m\overset{▪▪}{y}\\ \sum_{i=1}^{3}F_{izr}+\sum_{j=1}^{3}F_{izl}=0 \end{array}\right.\left(i\;j=1\sim 3\right)$$

where *F* is the force from the ground. The subscript *i* and *j* of *F* express the 1st leg, 2nd leg, and 3rd leg (foreleg, middle leg and hind leg, respectively). The subscript *x, y*, and *z* of *F* denote the component forces along the *x* axis, *y* axis, and *z* axis. The subscripts *r* and *l* of *F* denote the right leg and the left leg. The *x* and *y* are the position of the center of mass (CoM) of the locust.

We assume the forces from the right leg and the left leg are equal. At the same time, the force on the hind leg is much larger than that of foreleg and middle leg.

(2) $$\left\{ \begin{array}{l} F_{ixr}=F_{ixl}\\ F_{iyr}=F_{iyl}(i=1\sim 3)\text{and}\left\{ \begin{array}{l} F_{3xr}>>F_{1xr}+F_{2xr}\\ F_{3yr}>>F_{1yr}+F_{2yr} \end{array}\right.\\ F_{izr}=F_{izl} \end{array}\right.$$

when analyzing the take-off phase of locusts, the forces acting on the front and middle legs can be ignored. The Equation (1) can be simplified as:

(3) $$\left\{ \begin{array}{l} 2F_{3xr}=m\overset{▪▪}{x}\\ 2F_{3yr}-mg=m\overset{▪▪}{y} \end{array}\right.$$

The force *F*_3*yr*_ is provided by the ground along the coordinate axis *y*. When the leg of the locust and the ground do not slip, the force *F*_3*yr*_ multiplied by the friction factor is the maximum static friction force. Therefore, when the force in the *x* direction is less than or equal to the maximum static friction force, the leg of locusts does not slip. Then we obtained the following conditions.

(4) $$\{\begin{array}{c} F_{ixr}\leq \mu F_{iyr}\\ F_{ixl}\leq \mu F_{iyl} \end{array}and\mu \geq \frac{\overset{▪▪}{x}}{\overset{▪▪}{y}+g}$$

It is known that the friction factor between the ground and the legs of locusts affects the jumping performance of locusts. Therefore, the number of leg burrs of locust affects its jumping performance. The more the number, the better the jumping performance. When the number of leg burrs of locusts is relatively small, the friction between the ground and the legs of locusts is not enough to meet the acceleration condition of the body of locusts. At this time, the legs of locusts will slip to a certain extent, which will affect the jumping stability and jumping performance of locusts.

In the flight phase, the corresponding mathematical model was set up to analyze the influence of different flapping wings on the jumping performance of locusts. According to the work from Oertel (2010), we obtained the following equation:

(5) $$\begin{array}{l} F_{s}\left(t\right)=\frac{1}{2}C_{s}\rho_{k}S_{c}{\left[r_{s}\overset{▪}{\theta \left(t\right)}\right]}^{2} \end{array}$$

where *C*_*s*_ is the lift coefficient; ρ_*k*_is the air density; wing parameters *S*_*c*_ and *r*_*s*_ are the area of the wing and the distance length from the point to wing root; θ(*t*) is the flapping angular velocity of flapping wings. According to the formula (5), it can be concluded that when air density ρ_*k*_, lift coefficient *C*_*s*_ and wing flapping angular velocity θ(*t*) remain unchanged, the distance *r*_*s*_ from a certain point on the wing to the wing root and the area *S*_*c*_ of the wing decrease, which will lead to the decrease of lift provided by the insect flapping wing. It can be seen that after we clipped part of its wings, not only the area of the wings *S*_*c*_ decreased, but also the *r*_*s*_ decreased, then the lift provided by air to the locust wings would decrease, affecting the jumping height and distance. When the locust has no wings, that is, the area of the wings *S*_*c*_ and the *r*_*s*_ are both zero. At this time, the locust has no lift, but only the ground force when jumping, so the jumping height, and distance are greatly reduced.

**Figure 4:**
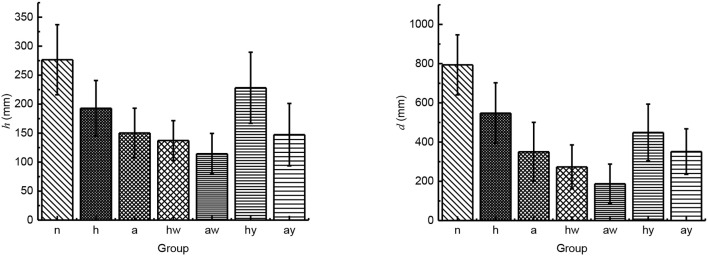
The influence of leg burs and wings on jumping performance (*n* stands for the group of locusts with normal leg burrs and normal wings; *h* stands for the group of locusts with half-leg burrs cut off and normal wings; *a* stands for the group of locusts without leg burrs and with normal wings; *hw* stands for the group of locusts without leg burrs and with half wings; *aw* stands for the group of locusts without leg burrs and without wings; *hy* stands for the group of locusts with all leg burrs and half wings; *ay* stands for the group of locusts with all leg burrs and without wings).

According to the experimental data, we analyzed the jumping performance of the above seven groups. We calculated the mean value and standard deviation of the jumping height and jumping distance with 10 times jump of each group. Through analyzing the results described in Figure 4, we found that the jumping height and the jumping distance of locusts with complete leg burrs were reduced to some extent after the part of leg burrs were cut off, and the jump height and the jump distance were further reduced when all of the leg burrs were cut off. Therefore, it is thought that under certain ground conditions, the number of leg burrs affects the jumping performance. The more the number of leg burrs, the better the jumping performance. When the number of leg burrs in is relatively small, the friction between the ground and the legs of locusts is not enough to satisfy the acceleration conditions of pushing locusts' bodies. At this time, locusts will slip on the ground to a certain extent, which will affect the stability and jumping performance of locusts.

Under the same number of leg burrs, we compared the results of wingless locusts with that of half-wing locusts, their jumping height and jumping distance decreased significantly. Compared with the complete-wing locust, the jumping height, and jumping distance of the half-wing locusts also decreased to a certain extent. Under the same wing length parameter, the jump height and jump distance of locusts without leg burrs are reduced to some extent. We compared the locusts without leg burrs to the locusts with complete leg burrs. So, we deduce that the flapping-wing movement of locusts also has a certain impact on jumping height and distance. Flapping-wing can help locusts improve jumping distance and jumping height.

Because biological experiments with animals vary from one individual to another and from one trial to another, and thus generate data with large variability, the jumping height, and distance values of locusts are generally different. The deviation of dispersion degree directly expressed by standard deviation is unreasonable. Therefore, the concept of coefficient of variation is introduced to represent the dispersion degree between different groups, so as to eliminate the influence of measurement scale and dimension on the measurement results. For this paper, the greater the value of the coefficient of variation, the worse the jumping stability. The coefficient of variation can be expressed as:

(6) $$\begin{array}{l} CV=\frac{SD}{MN}\cdot 100\% \end{array}$$

where *SD* is the sample standard deviation; *MN* is the sample mean.

**Figure 5:**
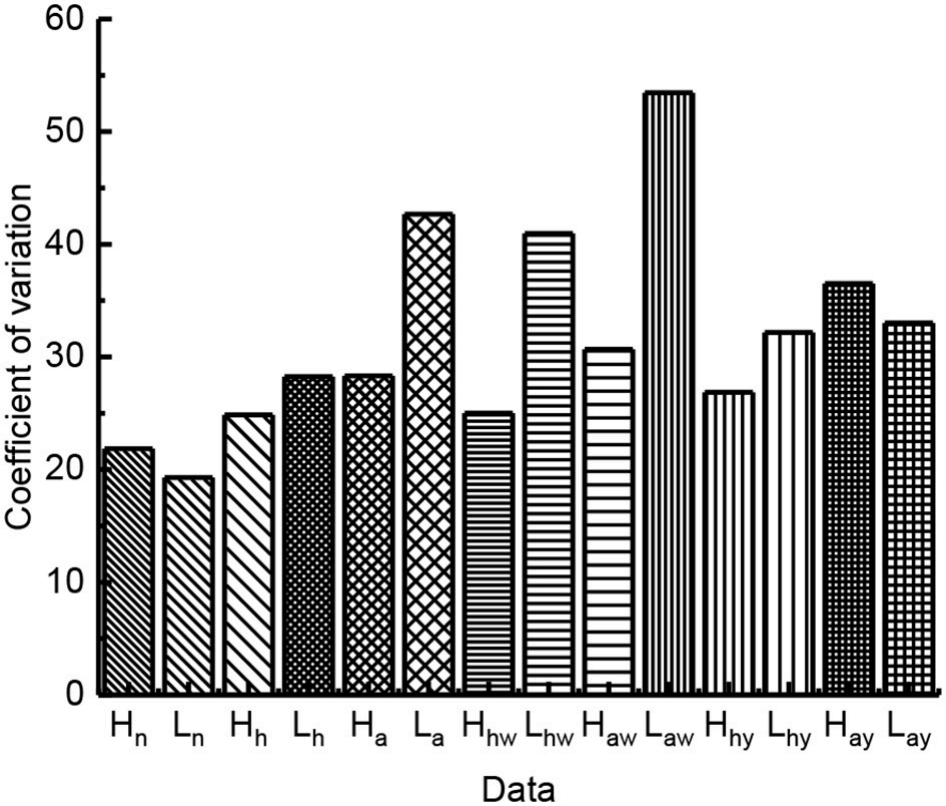
The variation coefficient of jumping performance.

As shown in Figure 5, the variation coefficient of jumping height and distance with different groups are different. The quantity of leg burr affects the *CV* of jump performance. The large value of the coefficient of variation indicates the instability of jumping. After comparing the value of *CV*, the results show the leg burr complete locust burr-free cut off part of the leg burr legs locusts with jumping height and distance of the coefficient of variation in turn increase, so the leg burrs affect stability, on the leg burr, the less the jump data dispersion degree that the greater the stability of the worse. Nevertheless, these factors include the following factors:

1. Individual heterogeneity results in the unconvincing experimental results. While animals vary from one individual to another and from one trial to another and thus generate data with large variability.
2. External stimulus is different and uncontrollable for locusts. Giving the same stimulus, the locusts may feel different incentives or they may be immune to stimuli over a long experimental period of time.
3. Surgery has different effects on individuals. The effect of the operation surgery for different locust is more or less different because of the operator and locust itself.

## Design of Locust-Inspired Robot

In the previous section, we carried out biological experiments and analyzed biological morphology and jumping performance. We preliminarily determined that the leg burrs and flapping wings of locusts have great influence on the jumping performance of living locusts. However, individual heterogeneity and uncontrollability of locusts resulted in the unconvincing experimental results. In this section, we proposed one robot prototype with flying, and jumping locomotion inspired by the locusts. This prototype has both flying and jumping locomotion based on metamorphic mechanism and lays a foundation for the study of robot inspired biology. In order to validate the deductions observed in the biological experiments and to remedy the shortcomings of biological experiments, we carried out some robot experiments for its consistency and controllability.

### Jumping Mechanism

**Figure 6:**
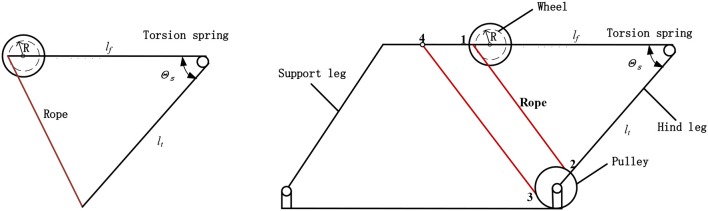
Jumping mechanism. *l*_t_ is the length of tibial leg, *l*_f_ is the length of the femoral leg, *R* is the effective radius of the pulley, and θ_*s*_ is the angle between *l*_*t*_ and *l*_*f*_.

Inspired by the jumping structure of locust hind legs, we selected the torsion spring as the energy storage element to mimic the muscle of locusts. And the rope was selected to act as a tendon in the locust's legs. As shown in Figure 6, the mechanism proposed in this paper has the advantage in torque output. Specifically, a natural lever ratio can be obtained through the rotating shaft and the radius of the rope pulley, which results in the increase of the torque. The transmission ratio between the torsion spring and the rope pulley can be expressed as follow:

(7) $$\begin{array}{l} K=R/\left(l_{t}\cos\frac{\theta_{s}}{2}\right). \end{array}$$

where *l*_t_ is the length of the tibia, *R* is the radius of the pulley, θ_*s*_ is the angle between *l*_t_ and *l*_f_. The actual transmission ratio is between 1/5 and 1/10, which increases the pulley's torque by 5–10 times. At the beginning of energy storage stage, the pulling force required is the smallest while θ_s_ is the largest. At this time, *K* is the largest (about 1/5), and the minimum gained multiple is obtained. At the moment before the end of the energy storage stage, the pulling force required is the largest while the θ_s_ is the smallest. It is known that *K* is the smallest (about 1/10), and the maximum gained multiple is obtained. In the process of energy storage stage, the pull force and the gained multiple are both increasing. Therefore, the minimum output torque of the motor can minimize to the greatest extent.

Another advantage of this design is that the torsion spring can reach a relatively large deformation angle about 100 degrees, which is more similar to the natural energy storage angle of the locust compared with other jumping robots. The gear system is driven by the motor, for the gear system can reduce the consumption of the motor and increase the output torque. Then the gear system drives the rope pulley to pull the rope and drive the rear leg to rotate. At the same time, torsion spring will be reversed and stores energy.

The motor is fixed on the central frame, and 6th gear is fixed coaxially with the rope pulley. The motor transmits the torque to the rope pulley through the speed-reduction gear system. One end of the rope is connected to the rope pulley, and the other end is connected to the 4th hole of the frame passing the movable pulley on the connecting rod which is at the end of the rear leg. And we made the rope 12 parallel to the rope 34. Because the movable pulley is installed on the connecting rod at the end of the hind leg, when the rope pulls the pulley, a torsion spring is given to the movable pulley through the connecting rod, which leads to saving half of the motor's force. According to the description of locusts' biological characteristics and the parameters of their limbs in chapter two, it can be seen that locust's femoral leg is almost as long as the tibial leg. So, we made *l*_*f*_ = *l*_*t*_ in the jumping mechanism we design. And in order to improve the stability of the robot, a parallelogram mechanism is used to synchronize the front support leg and the rear support leg. This design method can further rise the barycentre of the robot.

**Figure 7:**
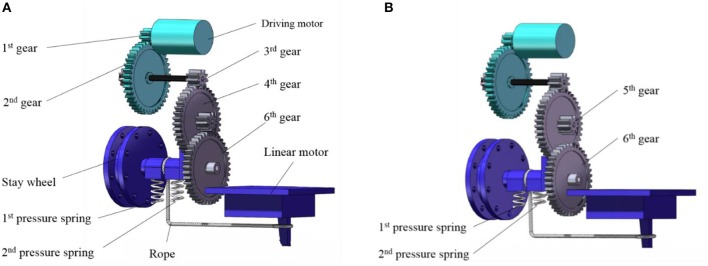
Jumping and metamorphic mechanism. **(A)** Energy storage state. **(B)** Trigger state.

As shown in Figure 7, when the energy storage stage begins, the main driving motor transmits the rotational motion to the pulley through the speed-reduction gear system. And the rope pulley drives the rope to shrink with the jumping leg rotating clockwise. When the torsion angle of the torsion spring is close to 100 degrees, the linear motor drives the axle sleeve to move downward. Resulting in 6th gear and 5th gear separate from each other instantly. At the same time, the linear motor stops operating.

Figure 7B is the trigger state. Because 6th gear suddenly loses meshing force, the rope wounded on the pulley will release rapidly under the force of torsional spring, then the jumping leg swings back quickly, and the robot will accelerate to complete the jumping motion. When the robot has landed on the land and is going to operate the next jump, the linear motor starts again and runs in another direction. Under the force of 1st pressure spring and 2nd pressure spring, 6th gear moves upward and meshes 5th gear again. Then torsion spring will store energy again to prepare for the next jump.

### Flying Mechanism

**Figure 8:**
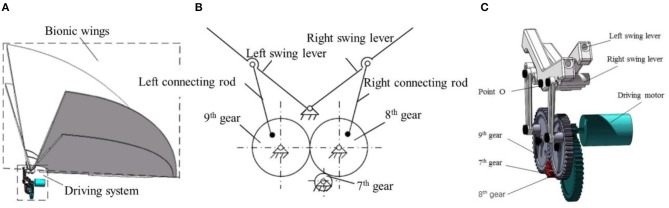
Flapping mechanism **(A)** overall layout of the flying mechanism; **(B)** the principle of gear-bar flying mechanisms; **(C)** 3D model of the flying mechanism.

The flapping mechanism we designed consists of two parts: biomimetic wings and driving mechanism. In consideration of the abundant motion and force transformation characteristics of the gear and connecting rod mechanism, we designed a pair of the symmetrical four-bar mechanism driving the wing support rod. The carbon fiber rod is inserted on the wing support rocker, and the wing is installed on the carbon fiber rod. When the wings begin to flap, the aerodynamic force produced by flapping up and down is unequal, so we designed the wings to flap up and down at a different speed. The mechanism is designed with quick return characteristic to make the flapping wing shoot up slowly and down rapidly. The flapping mechanism is shown in Figure 8. One end of the right connecting rod is connected with the 8th gear, the other end is connected with the swing rod. And both ends of the right connecting rod can rotate around the center of rotation, and the swing lever can rotate around the point *O* on the frame. The wing is mounted symmetrically on the left and right swing rods and the movements of the wings are the same.

### Metamorphic Mechanism and Coupling Mechanism

The metamorphic mechanism can transform the mechanism from one mechanism to another instantly, and the number of effective components or degrees of freedom will change. In order to design a robotic mechanism with the characteristics of instantaneous energy release from legs, the fixed posture of legs and wing flapping motion during the flight, we designed a metamorphic mechanism which can let the components merge and separate instantaneously. And we chose the rope to realize the instantaneous merger and separation between 5th gear and 6th gear. We also added a coupling module to couple the bionic jumping mechanism with the bionic flapping mechanism based on the aforementioned bionic jumping and flapping mechanism. For the linear motion characteristic of the linear motor, we used it to control the coupling between the jumping mechanism and the flapping wing mechanism.

From the coupling picture of gear system shown in Figure 7, it can be seen that the main drive motor, 1st gear and 2nd gear are shared through the metamorphic mechanism. 7th gear meshes with 8th gear, and the symmetrically distributed 8th gear and 9th gear drives the wings to flapping motion. 3rd gear on the other side transfers the torque to the pulley, which drives the leg to store and release energy, and realizes the coupling of jumping and flapping motion by a single main motor. In this paper, the pressure spring with both the characteristics of rigidity and flexibility is chosen for the coupling mechanism. The rigidity characteristic is used to provide 6th gear with support force and the flexibility characteristic is used to let 6th gear separate from 5th gear under the traction of the liner motor. In order to ensure that rope pulley can reverse under the spring force after 6th gear and 5th gear is separated from each other; we installed a sleeve with clearance fit between rope pulley and 6th gear. This sleeve not only plays an accurate positioning role, but also can quickly reverse under torsional spring force when being pulled.

Figure 7A shows the combined state of the metamorphic mechanism, that is, the combined meshing state of 5th gear and 6th gear. Figure 7B shows the separation state of the metamorphic mechanism, that is, the demeshing state of 5th gear and 6th gear, and the robot completes the jumping process.

## Robotics-Inspired Biology

In this section, we built the robot prototype with flying-jumping locomotion inspired by the locusts. Based on the idea of robotics-inspired biology, we carried out some preliminary experiments to validate the influence of leg burrs and flapping wings on the jumping performance, which remedied the shortcomings of the inconsistency, and uncontrollability in the biological experiments. Through the jumping test with the different number of leg burrs and the experiment with the static and flapping wing states, the effects of leg burrs and flapping wings on the jumping performance are revealed.

### Prototype of Locust Inspired Robot

**Figure 9:**
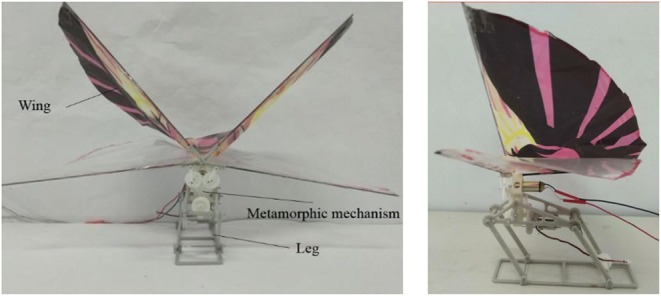
Prototype of bioinspired flying-jumping robot.

The robot prototype shown in Figure 9 was produced by the 3D printer, consisting of the gear-rod mechanism, gear transmission system, metamorphic mechanism, energy storage, and release mechanism. In order to make the robot achieve the best jumping performance, the weight of the robot should be as light as possible. The material we chose is presented in Table 1. We chose one hollow cup 820 DC motor with quite strong magnetic force and large torsion for aeromodelling which can achieve the non-load speed of 5,000 r/min with 3.7 V voltage and 0.1 A current.

**Table 1:** Materials and their density g/cm^3^.

**Body part**	**Material**	**Density**
Legs and external rack	PLA	1.26
Central support	ABS	1.05
Pulley	Nylon	1.15
Sole	PLA	1.26
Wing sport	PLA	1.26

### The Workflow of the Prototype

**Figure 10:**
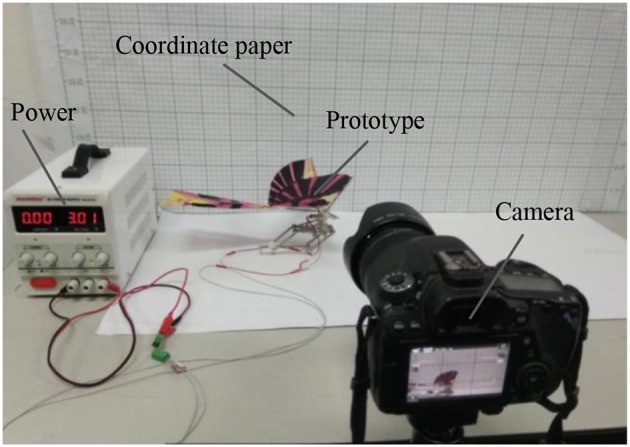
The experiment platform.

**Figure 11:**
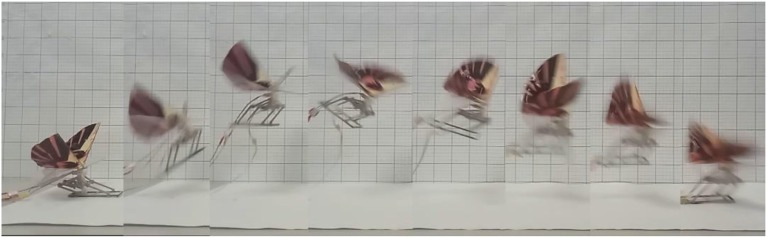
The motion sequences of flying-jumping locomotion with the proposed bioinspired robot.

Figure 10 is the experiment platform. Similar to the locomotion mode of the locusts, the designed robot has four stages in its flying-jumping process. The motion sequences of flying-jumping locomotion with the proposed bioinspired robot. Figure 11 is the motion sequences of flying-jumping locomotion with the proposed bioinspired robot. The first stage is called the jumping preparing stage. Firstly, the main motor begins to run, and the rope pulley is driven to rotate by the gear transmission system to wound the rope around the rope pulley. Then the tibia leg is driven by the rope to gradually approach the external frame. At the same, the torsion spring generates the torsion angle to store the energy needed for jumping. In this stage, the flapping wing mechanism flaps slowly. The second stage is called the jumping stage. In this stage the torsion angle of the torsion spring reaches 100 degrees, then the linear motor starts to run to separate the two gears from meshing, and the rope wound on the pulley is released instantaneously under the force of the torsion spring force, which makes the robot jump off the ground instantaneously. The third stage is called the flying stage. In this stage the linear motor stops running and keeps the gears five and six out of mesh to prevent legs from swinging, at the same time, the wings flap quickly to provide lift force. The fourth stage is called the landing stage. In this stage, the rotating speed of the main motor is reduced, with the wings flapping slowly. And the deformation of torsion spring absorbs the impact energy when touching the ground.

### Experimental Verification

In this section, we have carried out experiments to validate the influence of leg burrs and flapping-wing on jumping performance. We also compared the data that we got from the experiments on the prototype with the data of the biological experiments.

**Figure 12:**
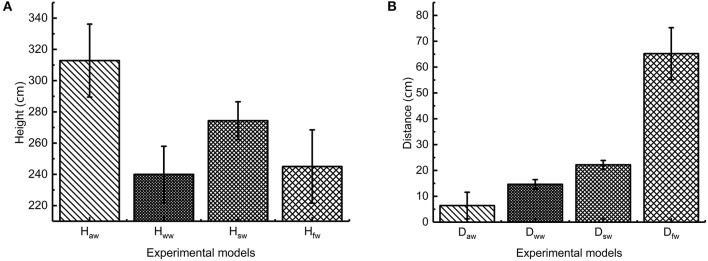
Jumping performance of locust bioinspired robot **(A)** jumping height **(B)** jumping distance *H*_*aw*_ stands for the average jumping height with burrs and without wings; *H*_*ww*_ stands for the average jumping height without burrs and wings; *H*_*sw*_ stands for the average jumping height with burrs and with static wings; *H*_*fw*_ stands for the average jumping height with burrs and flapping-wings; *D*_*aw*_ stands for the average jumping distance with burrs and without wings; *D*_*ww*_ stands for the average jumping distance without burrs and wings; *D*_*sw*_ stands for the average jumping distance with burrs and static wings; *D*_*ww*_ stands for the average jumping distance with burrs and flapping wings.

In the experiment on the influence of leg burrs on jumping performance, we removed the wings and conducted two groups of experiments. The robot in the first group jumps with burrs stuck to the foot and the robot in the second group jumps without burrs on its foot. The average value of the jumping performance in each group is presented in Figure 12. We can find that the maximum jumping height of the prototype without burrs is 330 mm; the longest jumping distance is 335 mm; the jumping height is about 3.1 times of the body length and the distance is about 3.1 times. The maximum jumping height of the prototype with burrs is 392 mm; the longest jumping distance is 305 mm; the jumping height is about 3.8 times of the body and the distance is about 3.2 times. It can be seen that leg burrs can increase jumping distance, jumping height, and jumping stability, which is consistent with the previous deduction derived from the biological experiment on the locusts.

In the experiment on the influence of flapping-wing on jumping performance, we conducted two groups of experiments. The first group jumps without wing-flapping motion. The second group jumps with wing-flapping motion. The average value of the jumping performance in each group is presented in Figure 12. We found that the maximum jumping height of the static wing is 270 mm. The longest jumping distance is 250 mm. The jumping height is about 2.6 times of the body length and the distance is about 2.6 times. The maximum jumping height of flapping wing prototype is 240 mm; the longest jumping distance is 780 mm; the height is about 2.3 times of the body length and the distance is about 8.2 times. According to the analysis of the experiment, it is not easy for the flapping-wing robot prototype to flip in the air during the jumping process, which greatly increases the jumping stability of the robot and reduces the damage to the robot. By comparing the results of data, it can be seen that the jump distance of the flapping-wing robot is greatly increased compared with that of the static wing robot, and the jumping height data are basically the same, which is consistent with the analysis of locust. Through the comparison of data results, it can be seen that compared with the robot with stationary wings, the jumping height, and jumping distance of the robot with flapping wings is greatly increased, and the flapping-wing flying motion exists in addition to the inertia force in the jumping and flying stage.

**Figure 13:**
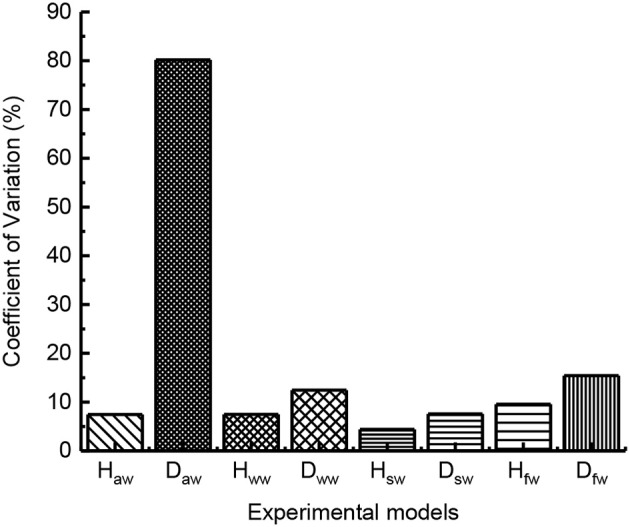
The coefficient of variation of jumping performance with robot *H*_*aw*_ stands for the average jumping height with burrs and without wings; *H*_*ww*_ stands for the average jumping height without burrs and wings; *H*_*sw*_ stands for the average jumping height with burrs and with static wings; *H*_*fw*_ stands for the average jumping height with burrs and flapping-wings; *D*_*aw*_ stands for the average jumping distance with burrs and without wings; *D*_*ww*_ stands for the average jumping distance without burrs and wings; *D*_*sw*_ stands for the average jumping distance with burrs and static wings; *D*_*ww*_ stands for the average jumping distance with burrs and flapping wings.

In order to analyze the influence of leg burrs and flapping wing on the jumping stability, according to the formula (6), the coefficients of variation were calculated based on the experimental results. As shown in Figure 13, we analyzed the coefficients of variation of the jumping performance including jumping height and jumping distance. The results dedicate that the coefficient of variation of the robot with leg burrs is relatively smaller than these of the robot without burrs. That is, the jumping locomotion of the robot with leg burrs is relatively stable, which is consistent with the results of biological experiments. Meanwhile, the robot with static wings has the smallest coefficient of variation compared with those experiments of robots (a) without burrs (b) with burrs (d) with flapping wings. This phenomenon indicates the wings greatly increases the jumping stability and reduces the damage to the robot. The jumping height data were basically equal, which was consistent with the analysis of biological experiments.

The analysis of jumping height data shows that the jumping process of the prototype in this paper is controlled by simple equipment, and the jumping motion of the robot occurs instantaneously. If the wings beat too early, the robot will generate a certain lift when it does not jump, which will cause the foot to slide on the ground, and affect the jumping height of the prototype. Through the experiment of the prototype, the validity of the previous deduction is verified.

## Conclusion

In this paper, we performed systematic experiments to understand the influences between the locomotors (both locust animals and bioinspired robots) and their jumping performance. The integration of hybrid inspired research contributed to the discovery of biology principles and the creation of bioinspired robot model, which also has proven powerfully in understanding the complex phenomena and predicting the design and control of robots.

We developed one simple but comprehensive robot prototype to validate and explain our observations from the biological experiments. Compared with other prototypes, the proposed robot inspired by locusts has multi-modal locomotion including jumping and flying based on metamorphic mechanism, which makes it perform the locusts' locomotion. We also discovered the sensitive dependence of locomotion performance on these leg burrs and flapping wings. We found that the number of leg burrs and the wing integrity has great influence the jumping performance, and the more the leg burrs are and the more complete the wings are the better jumping performance will be. Overall, our studies provided novel principles for bioinspired robots with flying-jumping locomotion, and at the same time, our robot-inspired experiment provides the insight to discover some principles behind the flying-jumping locomotion of biology.

At the current stage, the degree of freedom of the legs and flapping wings in our proposed prototype is limited. Multi-degree of freedom legs and the flexible wings will provide the potential to mimic more similar biological locomotion. The biological experiment is also hard to discover the principles of the coupling motion between the legs and the flapping wings during taking-off, air flight, and landing-off. In future work, we will have a tendency to concentrate on those issues based on the idea of hybrid inspired research.

## Data Availability Statement

The raw data supporting the conclusions of this manuscript will be made available by the authors, without undue reservation, to any qualified researcher.

## Author Contributions

DW and TG contributed the initial conception and obtained funding support. TG, SZ, and CZ conducted the experiments and data curation. DW, XM, and ZL wrote the original draft manuscript. DW, TG, and XM contributed to manuscript revision.

### Conflict of Interest

The authors declare that the research was conducted in the absence of any commercial or financial relationships that could be construed as a potential conflict of interest.
